# Inappropriate Use of Proton Pump Inhibitors for Stress Ulcer Prophylaxis in a Tertiary Care Hospital: A Cross-Sectional Study

**DOI:** 10.7759/cureus.83010

**Published:** 2025-04-25

**Authors:** Sardar Alam, Sadia Iqbal Qureshi, Ali Shakeel, Atika Javed, Nayab Sami, Bilal Ahmad

**Affiliations:** 1 Department of Gastroenterology, Bacha Khan Medical College, Mardan, PAK; 2 Internal Medicine, Ealing Hospital, London, GBR; 3 Internal Medicine, Ziauddin University, Karachi, PAK; 4 Medicine, Gujranwala Medical College, Gujranwala, PAK; 5 General Practice, Ever Care Hospital, Okara, PAK; 6 General and Internal Medicine, Khyber Teaching Hospital, Peshawar, PAK; 7 General Internal Medicine, NHS Ayrshire and Arran, Crosshouse, GBR

**Keywords:** guideline adherence, inappropriate prescribing, proton pump inhibitors, stress ulcer prophylaxis, tertiary care

## Abstract

Background: Although proton pump inhibitors (PPIs) are extensively used for acid-related disorders, their inappropriate administration for stress ulcer prophylaxis (SUP) in hospitalized patients remains a cause for concern. This study aimed to evaluate the extent of PPI misuse for SUP in a tertiary care setting, identify contributing factors, and assess adherence to clinical guidelines.

Methodology: A cross-sectional study was conducted over one year, including hospitalized patients receiving PPIs for SUP without documented indications. Data were collected through medical record reviews and physician questionnaires to determine prescription patterns, indications, and adherence to guidelines. Statistical analysis was performed using SPSS (IBM Corp., Armonk, NY, USA), employing chi-square tests and descriptive statistics.

Results: Among 274 patients, only 31.02% had appropriate indications for PPI use, while 68.98% received PPIs without meeting prophylactic criteria. Overall, 33.58% of prescriptions adhered to guidelines, whereas 66.42% deviated. The average duration of PPI use was 10.3 ± 4.7 days: 47.45% used PPIs for seven or fewer days, 31.02% for eight to 14 days, and 21.53% for more than 14 days. Guideline-based prescribing accounted for 35.77%, while 40.88% of prescriptions were attributed to defensive prescribing. Inappropriate PPI use was significantly associated with older age (p = 0.034) and ICU admission (p = 0.001).

Conclusion: PPI overuse for stress ulcer prevention is prevalent among hospitalized patients. Targeted interventions are needed to improve adherence to evidence-based guidelines and curb unnecessary prescribing, particularly in relation to defensive medical practices.

## Introduction

Proton pump inhibitors (PPIs) are among the most widely prescribed medications globally, primarily due to their effectiveness in treating acid-related conditions such as gastroesophageal reflux disease (GERD), peptic ulcer disease, and Helicobacter pylori infections [1,2]. In tertiary care settings, PPIs are frequently administered prophylactically to prevent stress ulcers; however, this widespread use has raised concerns about inappropriate prescribing practices [3].

Stress-related mucosal disease (SRMD) refers to gastrointestinal mucosal damage that can occur in critically ill patients, often due to physiological stress. While SUP with PPIs is recommended for high-risk individuals, such as those on mechanical ventilation for over 48 hours or with coagulopathy, their routine use in non-critically ill hospitalized patients is largely unnecessary and may contribute to adverse effects and increased healthcare costs [4,5].

Recent studies have linked inappropriate and prolonged PPI use to serious complications, including Clostridium difficile infections, osteoporotic fractures, chronic kidney disease, and deficiencies in micronutrients such as magnesium and vitamin B12 [6,7]. Despite these risks, PPIs are often prescribed without clear indications, sometimes continued unnecessarily after discharge, and commonly used in patients who lack significant risk factors for stress ulcers. This trend is particularly notable in tertiary care hospitals, where institutional habits and physician prescribing behaviors heavily influence clinical decisions [8,9].

Current guidelines from gastroenterology and critical care societies emphasize SUP only for critically ill patients with specific risk factors. Nevertheless, deviations from these recommendations remain common, often driven by defensive prescribing practices, institutional inertia, or lack of awareness [10,11]. Addressing these issues is crucial for enhancing patient safety, reducing avoidable adverse outcomes, and ensuring cost-effective pharmacotherapy in hospitalized patients.

Research objective

This study aimed to assess the prevalence of inappropriate PPI prescribing for stress ulcer prevention among hospitalized ICU and non-ICU patients in a tertiary care hospital, identify contributing factors, and evaluate adherence to established clinical guidelines.

## Materials and methods

Study design and setting

This cross-sectional study was conducted at the Bacha Khan Medical College, Mardan over a period of one year, from January 2023 to December 2023.

Inclusion and exclusion criteria

The research comprised hospital admissions of patients on PPIs for stress ulcer prophylaxis. Patients with a documented indication for PPI use such as Helicobacter pylori eradication treatment, peptic ulcer illness, or GERD were excluded.

Sample size

A total of 274 patients were included in the study using a convenience sampling method. This approach was chosen due to practical constraints, such as limited time and resources, and the readily accessible patient data from hospital records, which allowed for efficient data collection within the study period. The sample size was selected to provide a comprehensive overview of guideline adherence and prescription trends for PPIs used in stress ulcer prevention among hospitalized patients.

Data collection

Patient medical records, drug charts, and doctor prescriptions all helped compile the data. Documented were details on PPI indications, medication length, and adherence to therapeutic standards. Additionally, doctors were asked to evaluate their PPI prescription justification.

Prescriptions were evaluated against established clinical guidelines for stress ulcer prophylaxis. Inappropriate PPI use was defined according to the American Society of Health-System Pharmacists (ASHP) guidelines and international consensus recommendations, which specify indications such as mechanical ventilation >48 hours or coagulopathy in ICU settings. Prescriptions outside these indications, especially in non-critically ill patients without risk factors, were categorized as inappropriate.

A structured physician questionnaire was used to assess prescriber rationale (Appendix). It included items on the clinical reasoning behind PPI initiation, awareness of guideline criteria, and institutional prescribing practices.

Statistical analysis

SPSS (version 25; IBM Corp., Armonk, NY, USA) was used for data analysis of the gathered ones. Continuous data were summarized using mean and standard deviation; descriptive statistics including frequencies and percentages were used to characterize categorical variables. Chi-square tests were used to examine relationships between improper PPI usage and many patient- or physician-related variables. P-values less than 0.05 were significant.

Ethical approval

Ethical approval for the study was obtained from the institutional review board of Bacha Khan Medical College Mardan (877/BKMC). Informed consent was obtained from all participants before data collection to ensure ethical compliance and patient confidentiality.

## Results

Baseline characteristics of patients

Among the 274 patients, the mean age was 48.6 ± 15.2 years; 95 patients (34.67%) were under 40 years, 112 (40.88%) were between 40-60 years, and 67 (24.45%) were over 60. Gender distribution included 142 males (51.82%) and 132 females (48.18%). Regarding hospital admission status, 85 (31.02%) were ICU patients, while 189 (68.98%) were admitted to non-ICU wards. Common comorbidities included hypertension in 78 patients (28.47%), diabetes mellitus in 65 (23.72%), chronic kidney disease in 30 (10.95%), and liver disease in 22 (8.03%). Previous PPI usage was reported by 97 patients (35.40%). Detailed demographic and baseline characteristics of study patients are shown in Table 1.

**Table 1:** Baseline Characteristics of Study Patients PPI: proton pump inhibitor

Characteristic		Number of Patients (n)	Percentage (%)
Age (Years)	Age < 40 years	95	34.67
	Age 40-60 years	112	40.88
	Age > 60 years	67	24.45
	Mean ± SD	48.6 ± 15.2	
Gender	Male	142	51.82
	Female	132	48.18
Hospital Admission	ICU Admission	85	31.02
	Non-ICU Admission	189	68.98
Comorbidities	Hypertension	78	28.47
	Diabetes Mellitus	65	23.72
	Chronic Kidney Disease	30	10.95
	Liver Disease	22	8.03
	Previous PPI Use	97	35.40

PPI use

**Figure 1:**
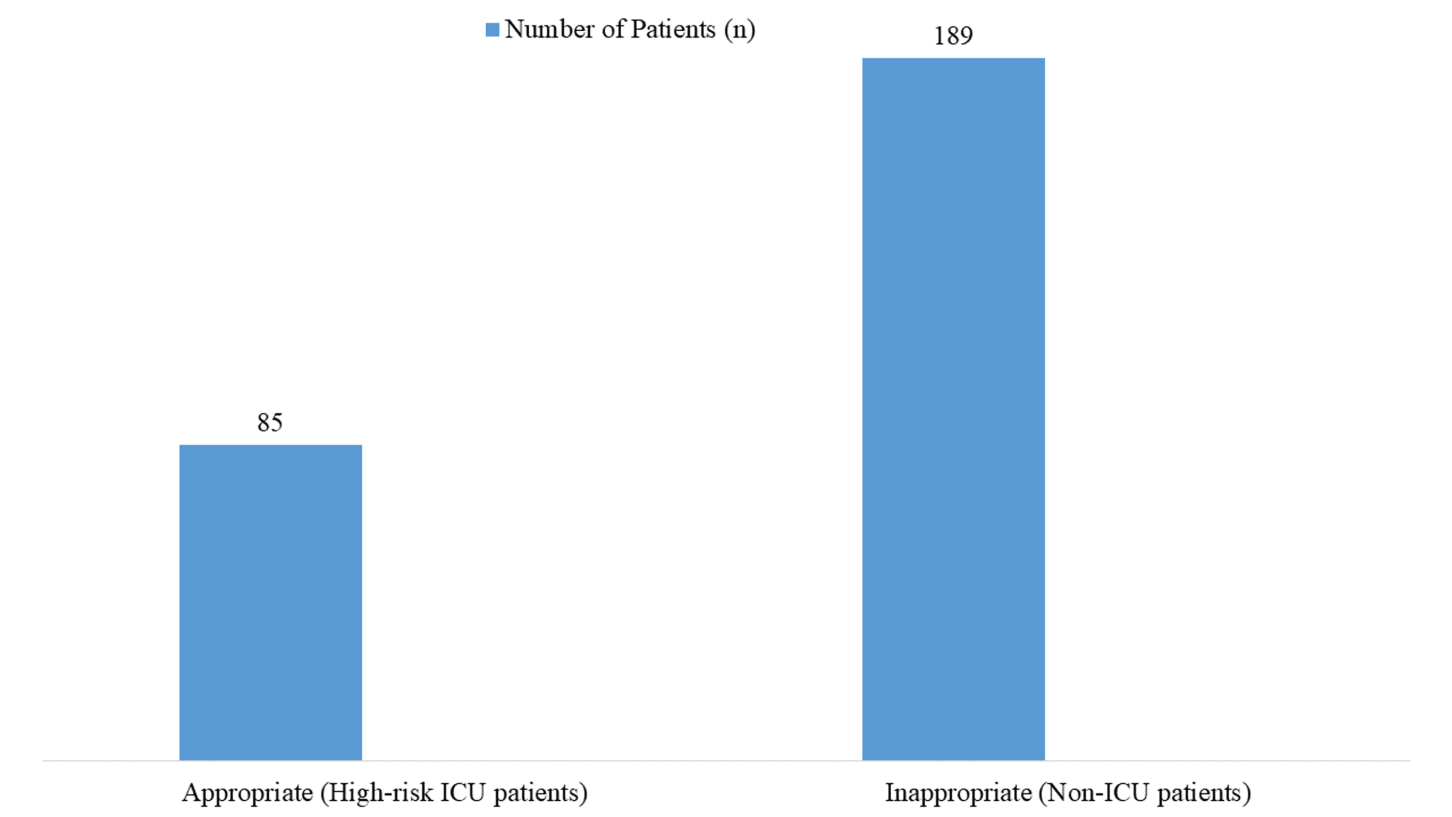
Appropriateness of Proton Pump Inhibitor (PPI) Prescriptions for Stress Ulcer Prophylaxis

Among the patients receiving PPIs for stress ulcer prophylaxis, only 85 (31.02%) had appropriate indications (high-risk ICU patients), while 189 (68.98%) received PPIs inappropriately, despite not meeting the criteria for prophylactic use (Figure 1).

Compliance with clinical guidelines

**Figure 2:**
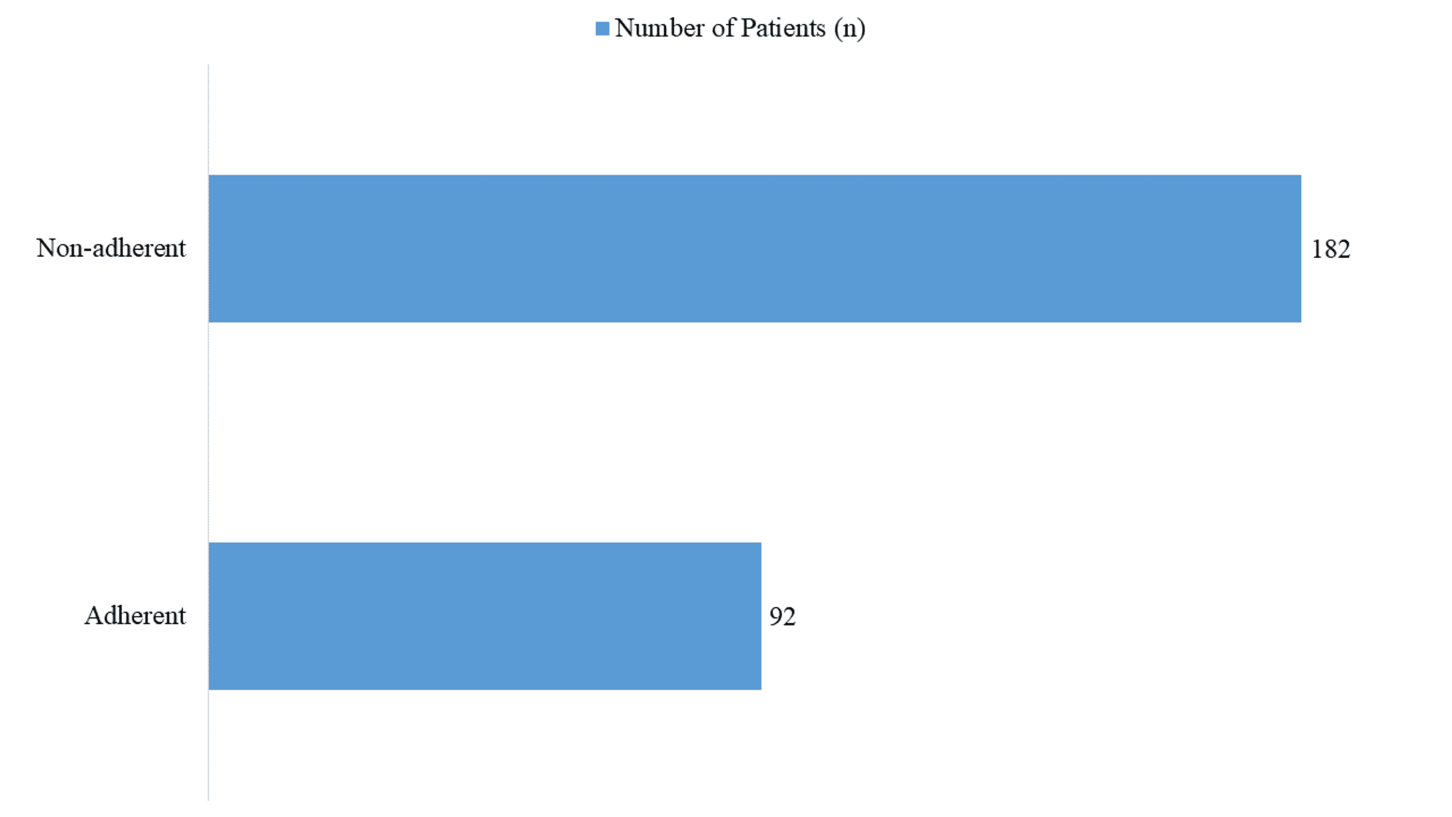
Compliance With Clinical Guidelines for Proton Pump Inhibitor (PPI) Use

Guideline adherence was observed in 33.58% of cases (n=92), whereas 66.42% (n=182) of prescriptions did not comply with recommended criteria, highlighting a significant deviation from evidence-based practices (Figure 2). The lack of adherence to clinical guidelines underscores the need for targeted educational interventions and policy reinforcement among healthcare providers.

Duration of PPI therapy

The mean duration of PPI therapy was 10.3 ± 4.7 days. Most patients (n=130; 47.45%) used PPIs for seven or fewer days, 31.02% (n=85) for eight to 14 days, and 21.53% (n=59) continued usage beyond 14 days, indicating prolonged and potentially unnecessary therapy in a substantial proportion (Table 2). The prolonged use observed in nearly one-fifth of patients raises concerns about increased risks of long-term complications such as hypomagnesemia and Clostridium difficile infections.

**Table 2:** Duration of Proton Pump Inhibitor (PPI) Therapy Among Hospitalized Patients

Duration of Use	Number of Patients (n)	Percentage (%)
≤7 days	130	47.45
8-14 days	85	31.02
>14 days	59	21.53
Mean Duration (Days)	10.3 ± 4.7	

Physician rationale for PPI prescription

**Figure 3:**
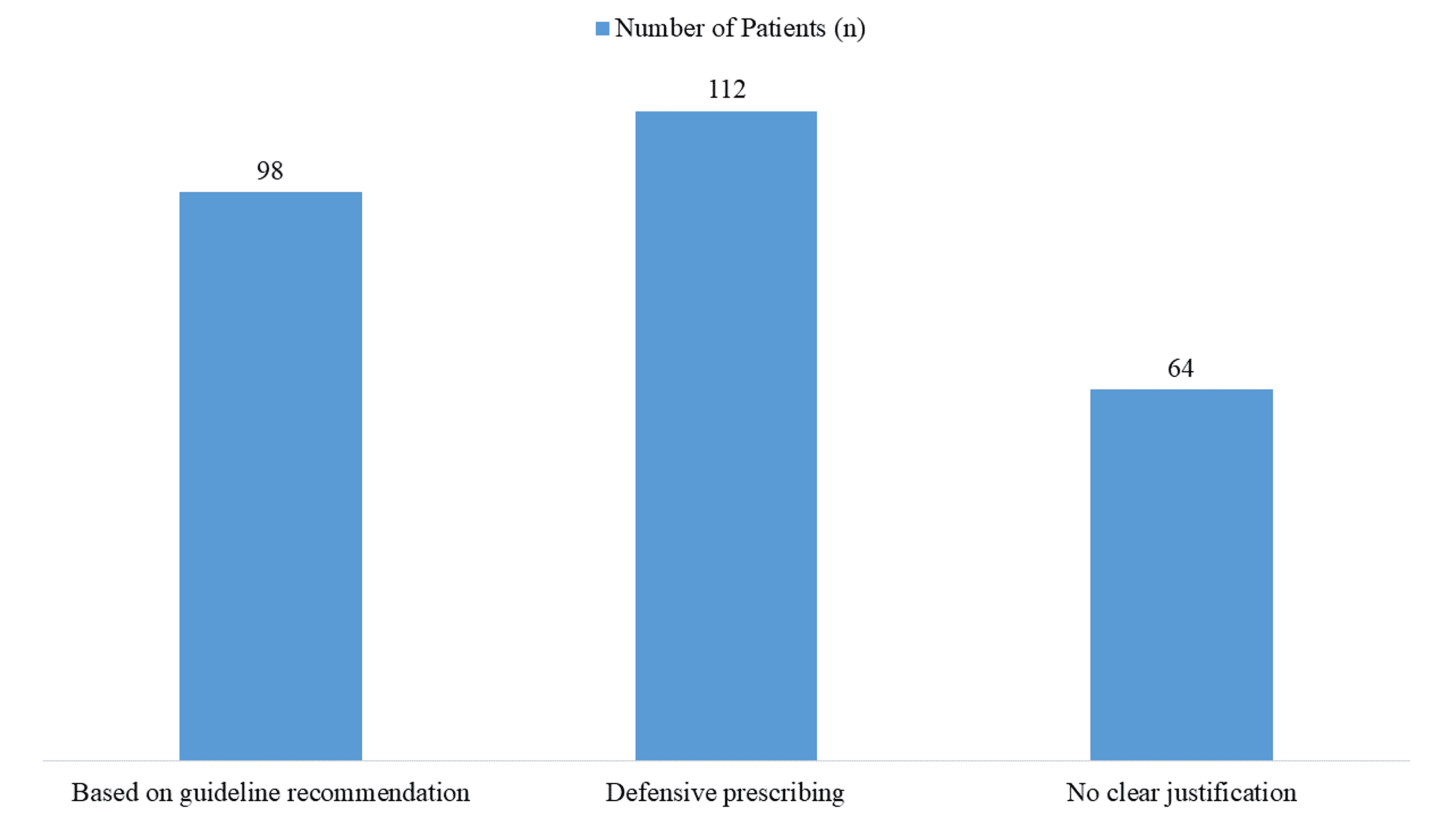
Physician Rationale for Proton Pump Inhibitor (PPI) Prescription

Among physicians prescribing PPIs, 35.77% (n=98) based their decision on guideline recommendations, while 40.88% (n=112) engaged in defensive prescribing. Notably, 23.35% (n=64) provided no clear justification for PPI use, reflecting gaps in appropriate decision-making (Figure 3). The high rate of defensive prescribing and undocumented rationale reflects a need for better clinical decision-support tools and documentation practices.

Association between patient factors and inappropriate PPI use

According to Table 3, chi-square analysis showed a significant association between inappropriate PPI use and age groups (χ² = 5.68, p = 0.034) as well as ICU vs. non-ICU admission status (χ² = 12.74, p < 0.001). However, gender was not significantly associated with inappropriate PPI use (χ² = 2.14, p = 0.143). These findings suggest that demographic and clinical settings influence prescribing behaviors, necessitating context-specific interventions to improve prescription appropriateness.

**Table 3:** Association Between Inappropriate Proton Pump Inhibitor (PPI) Use and Patient-Related Factors (Chi-Square Test Results)

Factor	χ² Value	p-Value
Age group (<40, 40-60, >60)	5.68	0.034*
Gender (Male vs Female)	2.14	0.143
ICU vs Non-ICU Admission	12.74	<0.001*

## Discussion

In tertiary care hospitals, the inappropriate use of PPIs for stress ulcer prophylaxis remains a significant concern, especially among non-critically ill patients. Our findings revealed that only 31.02% of prescriptions were justified based on accepted clinical guidelines, while 68.98% were administered without appropriate indications. These results align with previous studies highlighting widespread misuse. For instance, Savarino et al. [12] reported that over 60% of hospitalized patients received PPIs without valid reasons, leading to unnecessary drug exposure and increased healthcare costs. Similarly, a study conducted in a tertiary hospital in China found that nearly 70% of PPI prescriptions were inappropriate [13].

When analyzing adherence to guidelines, our study showed that 66.42% of prescriptions deviated from established criteria, while only 33.58% were compliant. A study in Ethiopia likewise reported that only half of physicians adhered to the American Society of Health-System Pharmacists (ASHP) guidelines, often prescribing PPIs defensively [14]. These findings point to the influence of institutional habits and lack of awareness as key contributors to PPI overuse, reinforcing the need for targeted educational and policy interventions.

Moreover, the duration of PPI use raised safety concerns. The mean treatment length was 10.3 ± 4.7 days, with 21.53% of patients receiving therapy for more than 14 days. Prolonged use increases the risk of serious complications, including Clostridium difficile infections, chronic kidney disease, and nutrient deficiencies such as hypomagnesemia and vitamin B12 deficiency [15]. Previous studies have shown that nearly half of PPI prescriptions continue beyond discharge unnecessarily. These findings support the importance of medication review and deprescribing protocols in tertiary care.

Prescribing patterns further revealed that 40.88% of physicians prescribed PPIs defensively - largely out of medicolegal concern - while only 35.77% adhered to evidence-based practices, and 23.35% had no clear rationale. This echoes other research indicating that fear of litigation often drives inappropriate prescribing. For example, one study found that nearly one-third of clinicians admitted to defensive PPI prescribing [16]. Implementing stewardship programs and targeted education may help reduce this trend.

Finally, statistical analysis showed significant associations between inappropriate PPI use and both patient age (χ² = 5.68, p = 0.034) and ICU vs. non-ICU status (χ² = 12.74, p = 0.001). Gender, however, was not a significant factor (χ² = 2.14, p = 0.143). These associations reinforce the need for focused efforts to curb overprescribing, particularly in non-ICU settings where stress ulcer risk is minimal [17,18].

Study strengths and limitations

This study’s focus on prescribing patterns in a tertiary care setting is a key strength, offering valuable insight into the extent of PPI use for stress ulcer prophylaxis. By including both ICU and non-ICU patients, the study provides a comprehensive assessment of prescribing trends and adherence to clinical guidelines. The use of chi-square analysis added statistical rigor by identifying significant associations between inappropriate PPI use and patient-related factors.

Several limitations should be acknowledged. The study was conducted at a single tertiary care hospital, which may limit the generalizability of findings to other settings with differing protocols or prescribing cultures. Reliance on medical records and physician-reported data may also introduce documentation bias. Importantly, the study did not assess clinical outcomes related to inappropriate PPI use, such as adverse effects or long-term dependency, which are essential for understanding the broader impact of misuse.

Future studies should include outcome measures and consider multicenter designs to validate and expand upon these findings. Such efforts would enhance generalizability and provide deeper insights into the clinical consequences of inappropriate PPI prescribing.

## Conclusions

This study highlights significant misuse of PPIs for stress ulcer prophylaxis in a tertiary care setting, with widespread non-evidence-based prescribing practices and poor adherence to clinical guidelines. Defensive prescribing and prolonged treatment durations emerged as key contributors to inappropriate use. Targeted interventions, such as physician education, guideline reinforcement, and the implementation of stewardship programs, are urgently needed. System-level strategies, including electronic health record (EHR) alerts and institutional stewardship committees, can play a pivotal role in reducing inappropriate prescriptions. Implementing such measures can improve patient safety, reduce unnecessary healthcare costs, and ensure more rational use of PPIs in hospitalized patients.

## Appendices

**Table 4:** Proton Pump Inhibitor (PPI) Prescription Questionnaire SUP: stress ulcer prophylaxis, EHR: electronic health record

Domain	Variable/Content	Question	Response Options
Clinical Justification	Risk Factors Present	Did the patient have any of the following risk factors justifying SUP? (e.g., mechanical ventilation >48h, coagulopathy, GI bleed history)	☐ Yes ☐ No (specify if yes)
Medication Details	PPI Type	Which PPI was prescribed?	☐ Omeprazole ☐ Pantoprazole ☐ Esomeprazole ☐ Other (specify)
Medication Details	Route of Administration	What was the route of administration?	☐ Oral ☐ Intravenous
Medication Details	Dose Appropriateness	Was the dose appropriate as per clinical guidelines?	☐ Yes ☐ No
Deprescribing	Prescription Review	Was PPI therapy reviewed or discontinued before discharge?	☐ Yes ☐ No ☐ Not Documented
Institutional Factors	Availability of Protocols	Are there existing hospital protocols for PPI use for SUP?	☐ Yes ☐ No ☐ Not Sure
Institutional Factors	Physician Awareness	Was the prescriber aware of clinical guidelines for SUP?	☐ Yes ☐ No ☐ Not Assessed
Institutional Factors	Access to Decision Support	Did the prescriber use any clinical decision support tool or alert system (e.g., EHR prompts)?	☐ Yes ☐ No ☐ Not Available
